# OREMPdb: a semantic dictionary of computational pathway models

**DOI:** 10.1186/1471-2105-13-S4-S6

**Published:** 2012-03-28

**Authors:** Renato Umeton, Giuseppe Nicosia, C Forbes Dewey

**Affiliations:** 1 Massachusetts Institute of Technology, 77 Massachusetts Avenue, Cambridge, MA 02139, USA; 2 Neurology and Department of Neurosciences, Mental Health and Sensory Organs, "Sapienza", University of Rome, S. Andrea Hospital-site, Via di Grottarossa 1035, Roma, RM 00189, Italy; 3 Department of Mathematics and Computer Science, University of Catania, Via A. Doria 6, Catania, CT 95125, Italy

## Abstract

**Background:**

The information coming from biomedical ontologies and computational pathway models is expanding continuously: research communities keep this process up and their advances are generally shared by means of dedicated resources published on the web. In fact, such models are shared to provide the characterization of molecular processes, while biomedical ontologies detail a semantic context to the majority of those pathways. Recent advances in both fields pave the way for a scalable information integration based on aggregate knowledge repositories, but the lack of overall standard formats impedes this progress. Indeed, having different objectives and different abstraction levels, most of these resources "speak" different languages. Semantic web technologies are here explored as a means to address some of these problems.

**Methods:**

Employing an extensible collection of interpreters, we developed OREMP (Ontology Reasoning Engine for Molecular Pathways), a system that abstracts the information from different resources and combines them together into a coherent ontology. Continuing this effort we present OREMPdb; once different pathways are fed into OREMP, species are linked to the external ontologies referred and to reactions in which they participate. Exploiting these links, the system builds species-sets, which encapsulate species that operate together. Composing all of the reactions together, the system computes all of the reaction paths from-and-to all of the species-sets.

**Results:**

OREMP has been applied to the curated branch of BioModels (2011/04/15 release) which overall contains 326 models, 9244 reactions, and 5636 species. OREMPdb is the semantic dictionary created as a result, which is made of 7360 species-sets. For each one of these sets, OREMPdb links the original pathway and the link to the original paper where this information first appeared.

## Background

**Figure 1:**
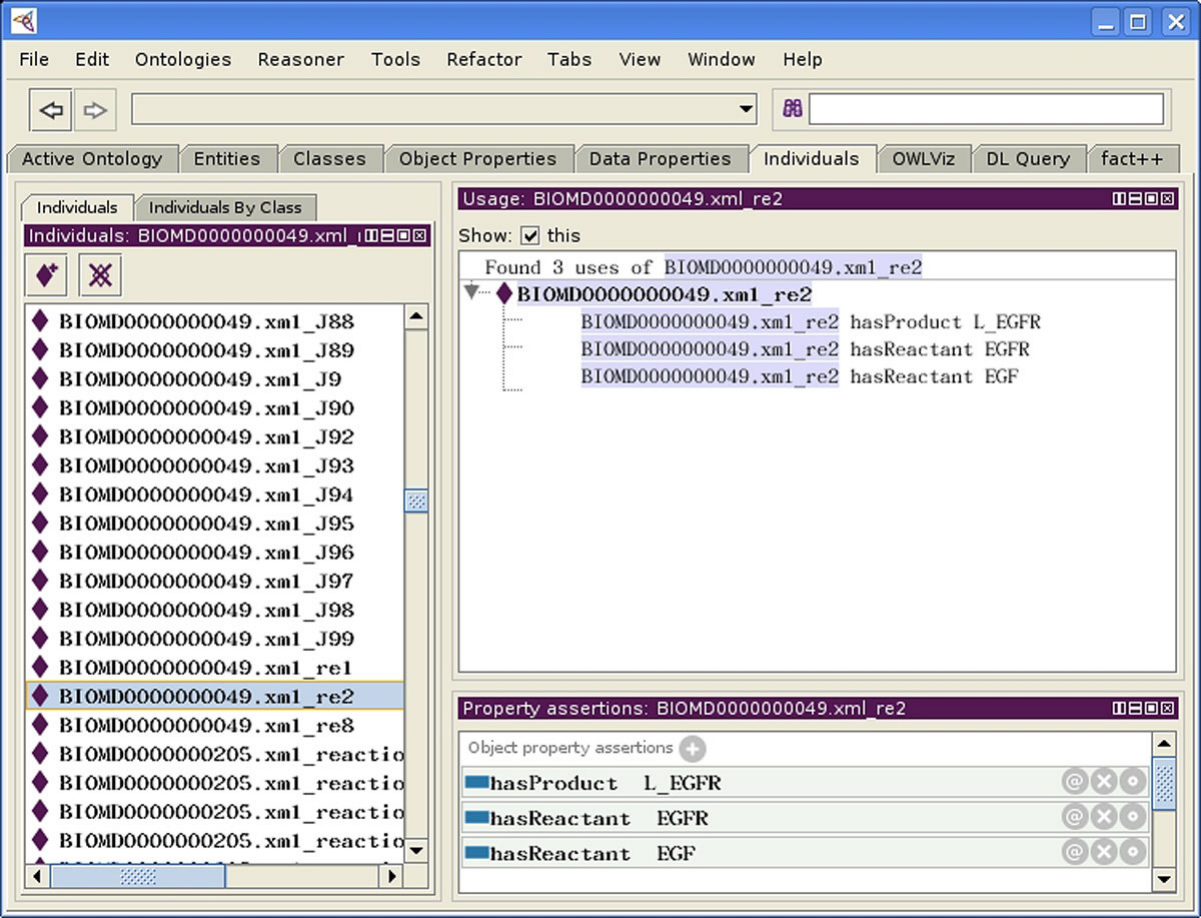
**OREMPdb queried in Protégé**. We selected OWL2 as export format and we adopted Protégé as default "Data Warehouse" for information storage, retrieval and reasoning; there OREMPdb, provides a single access point to the whole Biomodel.net database of curated models (326 computational pathway models, in 2011/04/15 release). Highlighted in the image: "BIOMOD0000000049.xml_re2", which is a reaction (left frame), and its associated triples (right frame), such as "BIOMOD0000000049.xml_re2 *hasProduct *L_EGFR". Overall the image presents Protégé showing the details of EGF-EGFR binding reaction (re2, in the model) in biomodel no. 49 [31], which has EGF and EGFR as reactants, and L_EGFR as product.

The information about molecular processes is expanding continuously and the descriptions are shared in the form of computable pathways. Biomedical ontologies are being created to provide a semantic context for the molecular species and reactions that they contain. Current advances in both topics suggest an information integration cycle based on shared knowledge-bases, but because of different languages (i.e., the data formats) spoken by the data sources and different abstraction levels, there is a lack of an overall frame capable of identifying overlaps and duplications [1]. One can envision searchable biological resources, such as the Gene Ontology (GO) [2], UniProt [3], ChEBI [4], KEGG [5], Reactome [6] and BioPortal [7], defining the biological context of the pathways in a machine-readable format. Substantial effort has been devoted to the creation of ontological resources which are publicly available, but there are semantic obstacles that inhibit their combined use. On the other hand, it is desirable to inform databases of computational pathway models, such as the BioModels.net collection, the CellML repository [8] and even specialist repositories [9-11], with the information contained in the curated molecular ontologies in a manner that can be used easily. Some syntactic conversions are available among pathway data-formats [12,13], and the state of the art for adjudication of the discrepancies between two SBML [14] models is SemanticSBML [15], which exploits machine-readable information and the user input to create a merged SBML model. Unfortunately, in the context of large-scale composite biological pathways, the merged-model approach is undesirable because it destroys the original component models and interrupts the curation process. For more than two SBML files, the tool must be run repeatedly with user-input, subjecting it to increasing human error, and suggesting that the order in which the models are aligned matters. An alternative approach based on the use of ontologies discerns when and on which topics models are a relevant part of the large-scale context. Where bio-ontologies are concerned, the state of the art is represented by BioPortal which provides uniform access to most of the biomedical ontologies through a single user-interface and advanced tools to query over biomedical data resources. As a matter of fact, there is still a large chasm between today's functionality and the true ability to use ontological data to inform molecular pathways. Additionally, there is a lack of strategies for the integration of quantitative biological sources based on different formats (e.g., SBML, CellML, and MML [16]); this means that although annotated by means of the same ontologies, computational pathway model repositories based on different formats cannot be unified preserving model dynamics. What is described here is a system that creates extended ontologies out of different biochemical information sources and provides path duplication detection, sharing, integration, and knowledge discovery over heterogeneous resources. This cross-format system, called OREMP (Ontology Reasoning Engine for Molecular Pathways) exports the extended ontologies (OREMPdb) in OWL2 format; the latter can be fed to Protégé [17], where the information can be then browsed, edited, and queried.We adopt Protégé (Figure 1) to give final users a platform where they can load the OREMPdb semantic dictionary, browse and edit its contents, and they can query it, finding bio-circuits of interest for them. On a separate track, we report that this system is currently employed in the MIT Cytosolve Project http://cytosolve.mit.edu where it is used to ensure correct parallel simulations.

## Methods

**Figure 2:**
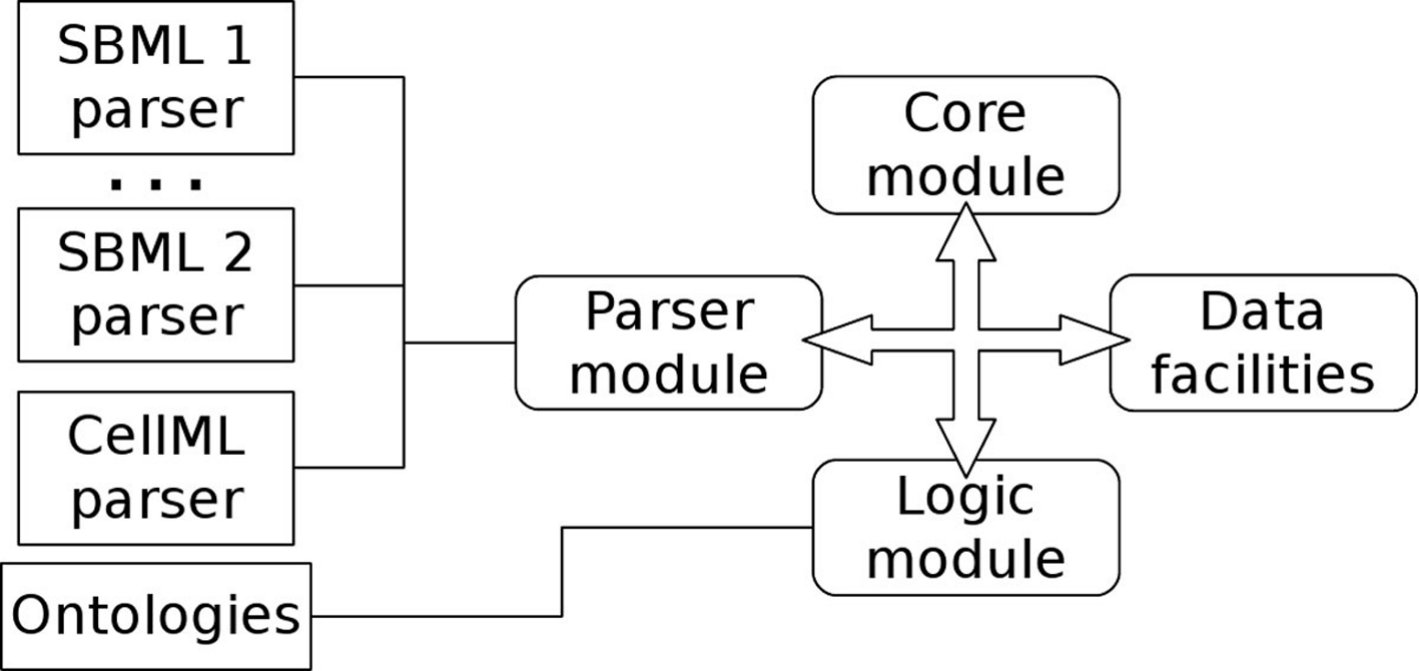
**OREMP system architecture**. System architecture: its components are integrated to work together preserving a flexible and easily extensible architecture. Each module has different versions used on the basis of job in progress (e.g., to parse an SBML Level 2 Version 1 file, it will be dynamically chosen the SBML 1 parser).

Biological processes can be effectively modeled by systems of ordinary differential equations (ODE). The equations themselves, along with their rate constants and initial conditions, need to be supplied to the ODE solver in a machine-readable format. A very popular standard format for describing the information content of models is the Systems Biology Markup Language (SBML) [14]. The SBML standard provides definitions for all model components that result in XML trees, that are machine-readable descriptions. Additionally, RDF parts inside SBML descriptions (e.g., annotations) can be expressed in XML and stored in XML repositories. A computational pathway models, or simply *model*, is a set of biochemical *species *whose evolution in time is determined by the *reactions *they participate in. These reactions, as well as the species, are specified in the SBML file. Reactions employ MathML [18] sections to specify how species concentration changes over time. Several ontologies [1] have recently been proposed to link species named in individual models to specific biological entities appearing in established curated biological information sources (e.g., GO, UniProt, ChEBI, and KEGG). A bio-ontology is an ontology where it is formally detailed how some life-science elements relate to each others. In order to step beyond simple syntactical translation, and exploit ontologies properly, we designed a system that merges the information from molecular pathways and curated biological ontologies into extended ontologies using a specific meta-format. The system is composed of the interchangeable and extensible components depicted in Figure 2; effectively, the information (e.g., species, reactions and references to ontologies) coming from heterogeneous resources is abstracted into an internal meta-format through these modular computational steps:

1. The *data access facility *collects information about multiple pathways and existing biological databases.

2. A *parser module *reads different file formats (i.e., XML, RDF, SBML, CellML, etc.) and extracts relevant information.

3. The *core module *assembles the knowledge, parsed from different sources, into a coherent ontology (based on the meta-format, cf. Table 1).

4. The *logic module *annotates all of the species from a collection of reactions and performs automated comparisons, identification of common species, and duplicate reactions.

**Table 1:** Main components of the minimalistic quantitative ontology

*Entity*	*has*
Annotation	type:STRING, uri:STRING, information:STRING.

Species	name:STRING, internalId:STRING, initialValue:REAL, inPathway:PATHWAY, hooks:SET_OF_ANNOTATIONS.

Kinetic reaction	internalId:STRING, kinetics:FORMULA, kineticParameters:SET_OF_PARAMETERS, inPathway:PATHWAY, reactants:SET_OF_SPECIES, catalysts:SET_OF_SPECIES, products:SET_OF_SPECIES, hooks:SET_OF_ANNOTATIONS.

Parameter	name:STRING, value:REAL.

Pathway	fullname:STRING, hooks:SET_OF_ANNOTATIONS.

Main components of the minimalistic quantitative MIRIAM-aware ontology used to abstract heterogeneous resources associated with biomolecular pathways. The format "attribute:REPRESENTATION" is used.

It is worth noting that different versions of each module can in fact be used. Employing a plugin pattern, an internal algorithm chooses the proper component implementation according to the current task (e.g., to read an SBML file, the system will invoke the SBML parser from its extensible list of parser modules). This means that whenever a new modeling format is introduced, a new parser (either provided as API together with the format definition, or developed in house by the OREMP team) can be connected to OREMP to interface with it as well. Similarly, different users can define different versions of the *core module*, for example, according to their understanding about how the knowledge coming from different pathways should be aggregated. This is of particular interest in domain-specific applications: according to different curators, different resources might be more valuable than others.

A key part of this approach is the designed meta-format. Around the latter the information is collated and merged together while preserving model identity; meaning that all reactions coming from all models are collated together, but, despite such fusion, each reaction preserves internally the link to the original model file it belongs to (Cf. the attribute *inPathway *in Table 1). This meta-format has been designed to embed the minimalistic and quantitative MIRIAM [19] information derived from different pathways. Model annotations are preserved and extended with supplemental quantitative data (coming from model reaction kinetics, for instance, and exported onto the attribute *kinetics *in the meta-format) to achieve a common description that can be represented as a single ontology. The structure of this ontology is presented in Table 1.

It is worth noting that, if we delete the link coming with the *inPathway *attribute, all of the elements abstracted in the meta-format can be disconnected from their original pathway and reasoned as if they all came from the same source. On the other hand, after this aggregate reasoning is performed, each conflict can be traced down to its source through the chain {*Species*|*Kinetic reaction*} ↔ *Annotation *↔ *Pathway*. This tunable abstraction level comes very handy when a pathway database has to be seen as a single source of information and its redundancies have to be flattened down. After interpreting different formats into the internal representation (the meta-format), another computational step is taken:

5. The *logic module *computes N-order species set-set reachability of all the reactions within the loaded and aligned models (connecting inter-model common species-sets, and filtering for instance the duplicate reactions, identified in the previous step, preventing then the creation of a multi-graph).

**Figure 3:**
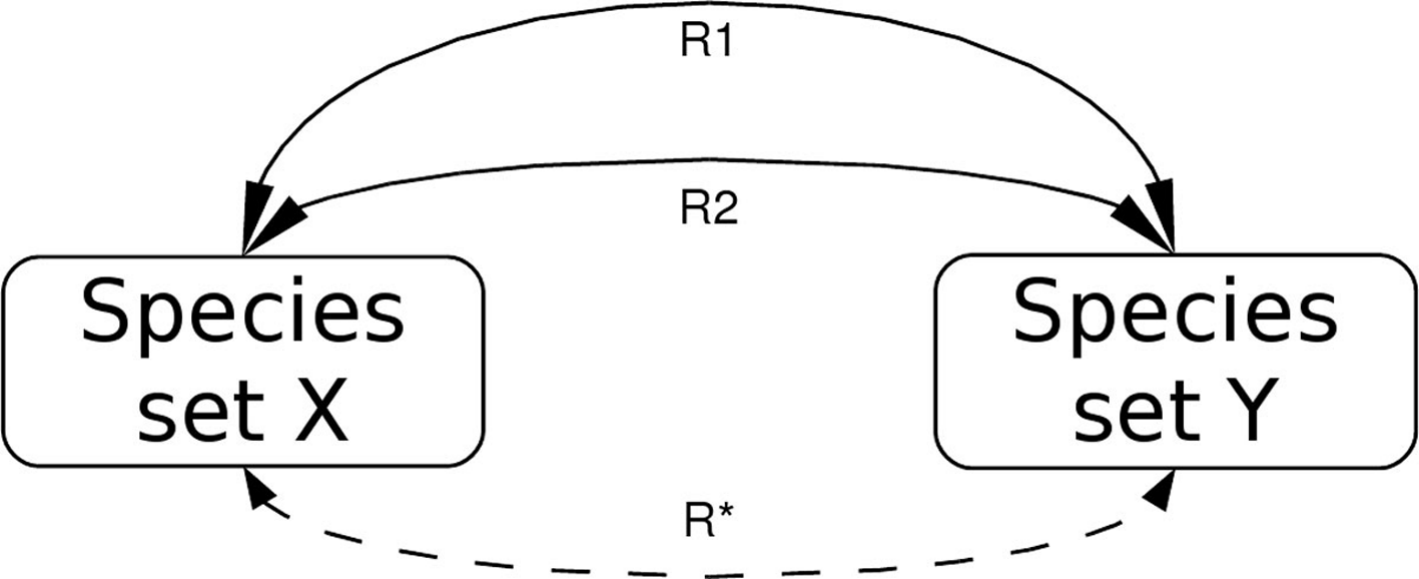
**First-order and N-order reaction overlaps**. This picture presents the two possible ways in which two species-sets can be connected: directly, by means of reactions such as R1 and R2, for instance, or indirectly, like for R*, which represents the fact that in order to move from species-set X to species-set Y, one or more additional species-sets have to be traversed. These concepts are used in the N-order reachability (duplicate reaction detection) among species-sets, which builds a reaction composition analysis by constructing a matrix which abstracts the overall species-set connectivity.

In empirical models, as said for model repositories, the detection of duplicates is extremely important because (for instance) a duplicate reaction may lead to erroneous results. The duplicates are revealed to the user, allowing individuals to retain editorial power over their models. It also assists researchers in understanding how the resulting models of their work fit into models produced by others. The N-order reachability (duplicate reaction detection) among species-sets builds a reaction composition analysis by constructing a matrix which represents a directed graph. Each vertex is a set of species and each edge is a reaction, which abstracts the overall species-set connectivity. This graph does not become a multi-graph for each set of duplicate reactions (first-order duplicate) because only one reaction is taken as a representative of its duplicate reactions. Through this reachability computation, a dictionary of potentially equivalent reaction compositions is built: candidate paths of the same starting and ending sets of species, but involving alternative intermediate paths. Figure 3 presents a case where first-order (N = 1, R1 and R2) and N-order (R*) duplicate reaction paths overlap: the dashed arc means that R* traverses more species-set apart from X and Y. The last computational step is the following:

The extended ontology is exported in OWL2 [20] and can be queried and edited by means of semantic tools.

## Results

The system has been tested in three real-world applications. (i) In a simple example, we demonstrated [21] the system's power to detect a first order duplicate reaction in the EGFR model [22] that has been factored up, but overlaps in one reaction, producing differences in quantitative results. Application (ii) consists in the fact that Cytosolve, which is a new computational environment for parallel simulation of multiple pathways, embeds a version of the OREMP system; there it is assigned to the task of identification of common molecular species and duplicated reactions with minimal human intervention. Last application (iii) is the combined analysis of the entire BioModels.net curated collection (currently 326 computational pathway models); OREMP has presented an aggregated view of the collection and brought to the identification of thousands of biological equivalent reaction chains; contextually, a dictionary of biological building blocks has been extracted. It is worth noting that we chose BioModels.net collection for the variety of models included and for its wide adoption; potentially, we could have imported two or more model databases, given that models contained were properly annotated. Relying on automatic annotation of models [23] perhaps we could work even with not annotated models in the future.

### OREMP in combining pathways for parallel solution

This system is embedded in the latest release of Cytosolve [24], which can be accessed at http://cytosolve.mit.edu. System contribution to the integration of computational pathway models is the detection of duplicated reactions among different models (in Cytosolve website, follow >Remote Solving, select or upload two or more models, >Align Models). No matter the models chosen for simulation, once the species are aligned, the system identifies duplication problems in the reaction-models. From the user point of view this process is transparent: he/she receives a warning message that details the duplicated reactions and is prompted to confirm conflict elimination, and to resolve any inconsistency in reaction kinetic rate constants. What follows is an example outline of the process that starts at http://cytosolve.mit.edu and moves from isolated pathways to their coherent parallel solution, employing OREMP to detect reaction duplicates.

**Figure 4:**
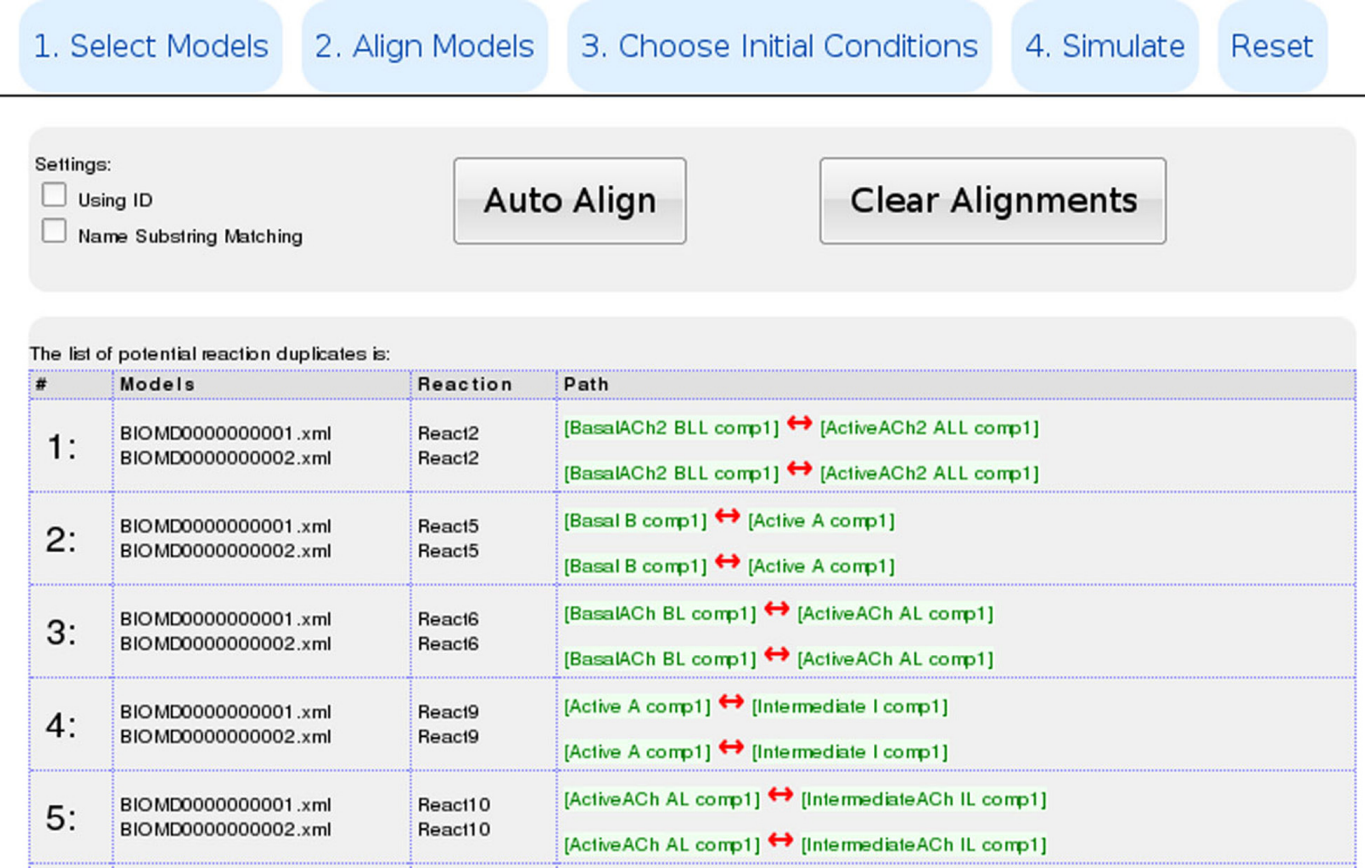
**OREMP employed inside Cytosolve**. Cytosolve, step 3: OREMP points out the overlaps among the two models.

**Figure 5:**
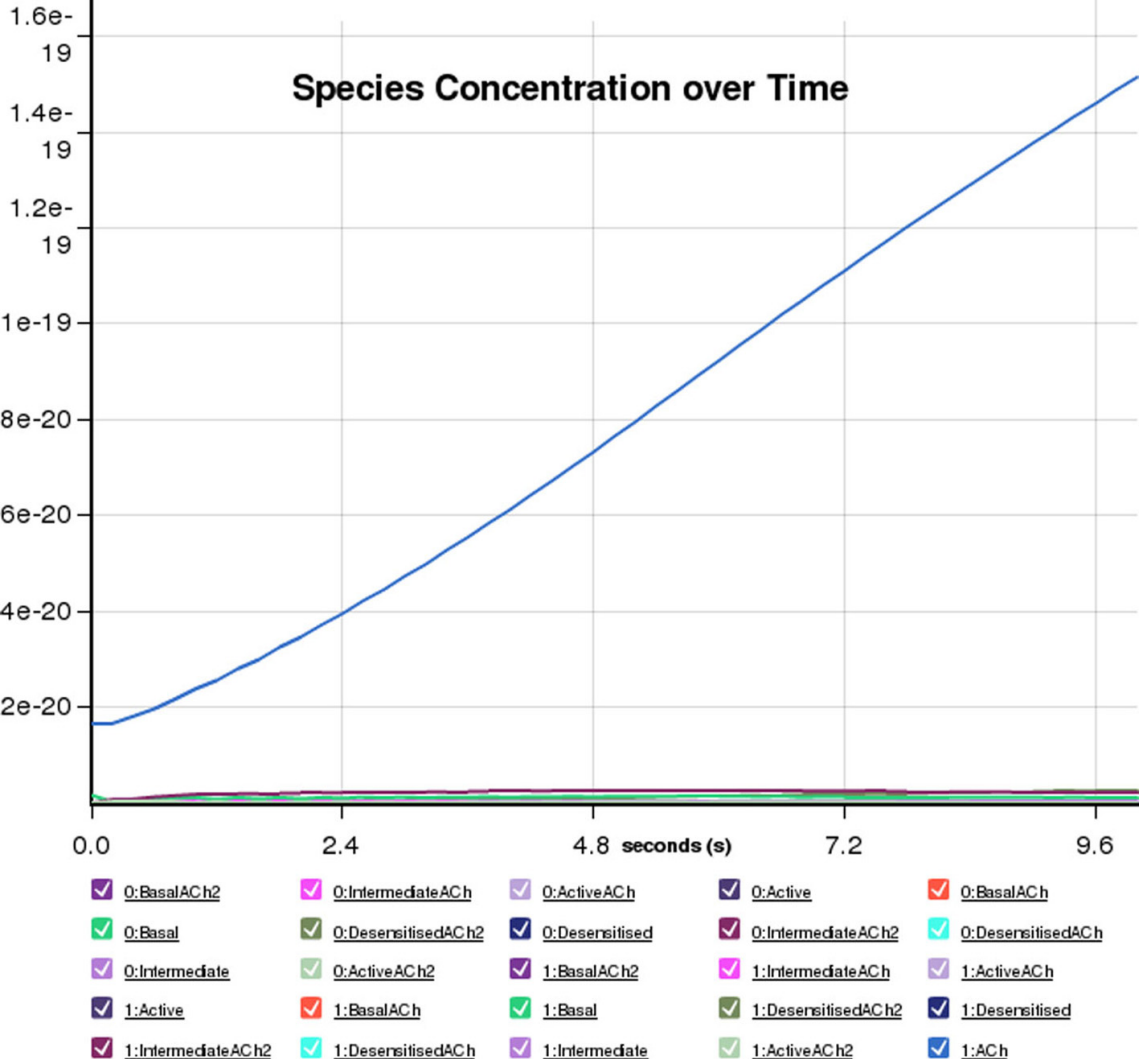
**Cytosolve simulation results**. Cytosolve, step 5: the simulation takes place and the results are visualized.

• Cytosolve, step 1, *Remote Solving*: Multiple Simulation begins

• Step 2, *Select Models*: models BIOMD..1 and BIOMD..2 are selected

• Step 3, *Align Models*: OREMP points out the overlaps among the two models, Figure 4

• Step 4, *Choose Initial Conditions*: the user silences the reaction in conflict and possibly re-uploads BIOMD..1

• Step 5, *Simulate*: the simulation takes place and the results are visualized, Figure 5

The prototype can be executed on groups of arbitrary models within Cytosolve homepage, repeating Steps 1-5 above, and choosing Upload as model source.

### OREMP in querying large, independent sources of pathways

The system has been tested against the entire BioModels.net curated collection [25] that contains 326 computational pathway models (release of 2011/04/15, which is the latest official release, at the time of paper writing). The result of the analysis is an overall view of the database and a list of about 500 groups of overlapping reactions, employing 7360 species-sets. This analysis took about *one minute *on a dual-core 2 GHz Intel CPU. The previously described knowledge-discovery-step involving N-order reachability has been taken on these resources as well. For each species configuration in the database, all alternative circuit paths have been computed. This took about *2 hours *on a quad-core 2 GHz AMD CPU and resulted in a dictionary of thousands "biological equivalent" circuits (i.e., equivalent reaction compositions). The latter dictionary namely OREMPdb, is composed of:

• An ordered dictionary of pathway building blocks

• The list of equivalent reactions overall used

• All of the potentially equivalent N-order reaction compositions

With this method the observed edge/vertex ratio for the BioModels.net curated DB is 1.19, similarly to other biological pathway databases - HumanCyc DB [26] has a ratio of 1.01 and EcoCyc DB [27] one of 1.25 [28]. These ratios suggest that the analysis we performed on BioModels.net reveals a connectivity density comparable with other biological pathway databases. A basic example of pathway building blocks extracted from the BioModels DB processing follows; this example includes only one species in each species-set. In the context of another EGFR model [29] (i.e., MAP kinase cascade activated by surface and internalized EGF receptors), as detailed in biomodel no. 19 in [22], the system detected that the *EGF *- *EGFR^2 ^*- *GAP *- *Shc *species can directly become EGF - EGFR^2 ^- GAP - Shc* or, alternatively, the former can first become *EGF *- *EGFRi^2 ^*- *GAP *- *Shc*, then EGF - EGFRi^2 ^- GAP - Shc*, and finally EGF - EGFR^2 ^- GAP - Shc*. This is just one and very simple example of the N-order analysis. In general, given two species-sets, OREMPdb provides all of the alternative ways to traverse from one to the other; as stated in the coming section, Protégé is the ideal tool to browse and query OREMPdb and perform this kind of queries.

### Ontologies from pathways: practical advantages

From a logic point of view, the system is constructed of three layers. The bottom layer represents the original biochemical pathways, read in their primitive format (such as SBML and CellML). The second layer abstracts (through the work-flow 1-6 detailed in §Methods) the pathways into a minimalistic and quantitative meta-format (sketched in Table 1) that includes MIRIAM components. Annotations are preserved and extended with additional quantitative data to achieve a common description that can be represented as a single ontology. It is at this level that the extended ontology is primarily created. Entities and relations created in this manner are homogeneous in the ontological sense. This implies that several collections of annotated pathways can be combined in OREMPdb while maintaining a common semantic, meaning that the following advantages are achieved:

#### Sharing

Despite disparate initial data formats, the biochemical information described in each pathway is now homogeneously represented in OWL2. This enables the direct reuse of componets (such as species or reactions) coming from different sources.

#### Integration

The system ensures a consistent merging of the resources, automatically aligning the species and showing the end-user possible duplications among reactions in the different pathways.

#### Knowledge discovery

Once the species alignment is done and duplicate reaction have been detected, the N-order reachability step is taken: for each reaction in each pathway the set of "alternative circuits" is computed. This means that given an arbitrary number of pathways, the system will identify all of the alternative ways to traverse from a species-set to another, employing all of the available reactions. In the last layer, all the information gathered is exported in OWL2, and Protégé is employed to visually edit, compare, and finalize the biochemical information exported. Protégé query interface allows the user to formulate "semantically-enabled" queries that were impractical when dealing with previously heterogeneous, unaligned models, Figure 1.

## Discussion

We described OREMP, but other tools are available too in the context of data integration; a major distinction in this context has to be done between those softwares that filter out the dynamics of computational pathway models, such as [30], and those that are kinetics-oriented. In this section is detailed the comparison with a leading one which is, as our work, kinetics-aware: SemanticSBML [15]. SemanticSBML provides the state of the art tools to obtain a monolithic merged model starting from different molecular pathways. Where Cytosolve is concerned, one key component of its approach is the fact that it does not produce a monolithic model. This preserves the curation process of independent models and allows independent research laboratories to continue investigation and improvement of their own model without being forced to prematurely publish an authoritative merged resource; the independent curation process is preserved by maintaining the pathway identity, since the primitive element-pathway network is not destroyed by integration. Basically, this approach is different from SemanticSBML because it provides the user the opportunity to exploit his/her understanding to define a consistent method of knowledge integration across ontologies. Another point of strength is the fact that once the system has read all of the 326 models from BioModels.net curated collection and the pathway building block dictionary is written (feeding step), the end-users can exploit this functionality to accelerate their research by taking advantage of other modelers efforts simply by consulting the OREMPdb dictionary. By specifying the initial and ending set of species, modelers can use the building block dictionary to gain ideas about how other people investigated and modeled a similar problem and how cross-pathway reactions could be composed to fit their needs. The experiment detailed in previous sections provided an interesting overview of the BioModels.net collection that brought also the following achievement: from the prospective of those who curate collections of biochemical pathways, this framework can be used to find inconsistencies and redundancies within their repository since the system highlights common bricks shared among multiple models.

## Conclusion

To our knowledge, this is the first time that the information coming from different biological data sources has been aggregated into a single quantitative ontology. OREMP application can combine several pathways, merge and combine pathways, or revert to the original pathways, and inspect single-model details and employ external biological annotations. The system is independent of the different file formats in which the pathways are written and contains an extensible collection of parser modules. These advantages are fully transferred to Cytosolve, which employs this system to ensure semantically-correct parallel simulations. We selected OWL2 as export format and we adopted Protégé as default "Data Warehouse" for information storage, retrieval and reasoning; there OREMPdb, provides a single semantic access point to the whole Biomodel.net database of curated models (currently 326 models, release of 2011/04/15).

## List of abbreviations used

API: Application Programming Interface; BioModels: European Bioinformatics Institute Biological Model Database; BioPortal: National Center for Biomedical Ontology Biology Portal; CellML: Cell Markup Language; ChEBI: Chemical Entities of Biological Interest; EcoCyc: Encyclopedia of Escherichia coli K-12 MG1655 Genes and Metabolism; EGF: Epidermal Growth Factor; EGFR: Epidermal Growth Factor Receptor; GAP: RAS p21 Protein Activator 1; GO: Gene Ontology; HumanCyc: Encyclopedia of Homo sapiens Genes and Metabolism; KEGG: Kyoto Encyclopedia of Genes and Genomes; MAP kinase: Mitogen-Activated Protein kinase; MathML: Mathematical Markup Language; MIRIAM: Minimum Information Required In the Annotation of Models; MML: Mathematical Modeling Language; ODE: Ordinary Differential Equation; OREMPdb: Ontology Reasoning Engine for Molecular Pathways database; OREMP: Ontology Reasoning Engine for Molecular Pathways; OWL2: Web Ontology Language, version 2; RDF: Resource Description Framework; SBML: Systems Biology Markup Language; SHC: SHC Transforming Protein 2; UniProt: Universal Protein Resource; XML: Extensible Markup Language.

## Competing interests

The authors declare that they have no competing interests.

## Authors' contributions

RU developed the software with the supervision of GN, CFD conceived the work and supervised the entire project development. All authors read and approved the final manuscript.
